# Accuracy and tolerability of self-sampling of capillary blood for analysis of inflammation and autoantibodies in rheumatoid arthritis patients—results from a randomized controlled trial

**DOI:** 10.1186/s13075-022-02809-7

**Published:** 2022-05-25

**Authors:** Johannes Knitza, Koray Tascilar, Nicolas Vuillerme, Ekaterina Eimer, Paul Matusewicz, Giulia Corte, Louis Schuster, Timothée Aubourg, Gerlinde Bendzuck, Marianne Korinth, Corinna Elling-Audersch, Arnd Kleyer, Sebastian Boeltz, Axel J. Hueber, Gerhard Krönke, Georg Schett, David Simon

**Affiliations:** 1 Department of Internal Medicine 3, Rheumatology and Immunology Friedrich-Alexander University Erlangen-Nürnberg and Universitätsklinikum Erlangen, Erlangen, Germany; 2 Deutsches Zentrum für Immuntherapie, Friedrich-Alexander University Erlangen-Nürnberg and Universitätsklinikum Erlangen, Erlangen, Germany; 3 University Grenoble Alpes, AGEIS, Grenoble, France; 4 Institut Universitaire de France, Paris, France; 5 LabCom Telecom4Health, Orange Labs & Univ. Grenoble Alpes, CNRS, Inria, Grenoble INP-UGA, Grenoble, France; 6 Thermo Fisher Scientific, Freiburg, Germany; 7 Deutsche Rheuma-Liga Bundesverband e. V, Bonn, Germany; 8 Division of Rheumatology, Nürnberg Hospital, Paracelsus Medical University, Nürnberg, Germany

**Keywords:** Self-sampling, Capillary blood, Rheumatoid arthritis, Disease activity

## Abstract

**Background:**

Rheumatoid arthritis (RA) requires early diagnosis and tight surveillance of disease activity. Remote self-collection of blood for the analysis of inflammation markers and autoantibodies could improve the monitoring of RA and facilitate the identification of individuals at-risk for RA.

**Objective:**

Randomized, controlled trial to evaluate the accuracy, feasibility, and acceptability of an upper arm self-sampling device (UA) and finger prick-test (FP) to measure capillary blood from RA patients for C-reactive protein (CRP) levels and the presence of IgM rheumatoid factor (RF IgM) and anti-cyclic citrullinated protein antibodies (anti-CCP IgG).

**Methods:**

RA patients were randomly assigned in a 1:1 ratio to self-collection of capillary blood via UA or FP. Venous blood sampling (VBS) was performed as a gold standard in both groups to assess the concordance of CRP levels as well as RF IgM and CCP IgG. General acceptability and pain during sampling were measured and compared between UA, FP, and VBS. The number of attempts for successful sampling, requests for assistance, volume, and duration of sample collection were also assessed.

**Results:**

Fifty seropositive RA patients were included. 49/50 (98%) patients were able to successfully collect capillary blood. The overall agreement between capillary and venous analyses for CRP (0.992), CCP IgG (0.984), and RF IgM (0.994) were good. In both groups, 4/25 (16%) needed a second attempt and 8/25 (32%) in the UA and 7/25 (28%) in the FP group requested assistance. Mean pain scores for capillary self-sampling (1.7/10 ± 1.1 (UA) and 1.9/10 ± 1.9 (FP)) were significantly lower on a numeric rating scale compared to venous blood collection (UA: 2.8/10 ± 1.7; FP: 2.1 ± 2.0) (*p*=0.003). UA patients were more likely to promote the use of capillary blood sampling (net promoter score: +28% vs. −20% for FP) and were more willing to perform blood collection at home (60% vs. 32% for FP).

**Conclusions:**

These data show that self-sampling is accurate and feasible within one attempt by the majority of patients without assistance, allowing tight monitoring of RA disease activity as well as identifying individuals at-risk for RA. RA patients seem to prefer upper arm-based self-sampling to traditional finger pricking.

**Trial registration:**

DRKS.de Identifier: DRKS00023526. Registered on November 6, 2020.

**Supplementary Information:**

The online version contains supplementary material available at 10.1186/s13075-022-02809-7.

## Introduction

Rheumatoid arthritis (RA) is one of the most common autoimmune diseases [1] causing chronic inflammation and loss of function. Since RA is a chronic disease, patients require a life-long care with close monitoring of disease activity. However, as there is an increasing shortage of specialized health care for patients with rheumatic and musculoskeletal diseases [2], patients often face challenges of receiving timely and adequate on-site care. This shortage has lately been exacerbated by the COVID-19 pandemic, in which a temporary reduction in appointment slots has been observed [3].

In addition to the need for remote monitoring of disease activity in patients with RA, it is also important to detect the disease at an early stage, i.e., identifying patients at-risk for developing RA, since delayed diagnosis is associated with joint damage, loss of function, and lower treatment efficacy [4]. Being “at risk of RA” is closely related to the presence of antibodies against cyclic citrullinated peptides (anti-CCP) or rheumatoid factor (RF IgM)). At present, however, assessment of the presence of RA-related autoantibodies is exclusively done by healthcare professionals via venipuncture.

Remote monitoring of disease activity may substantially improve clinical care [5]. A challenge for comprehensive monitoring of diseases, such as RA is the blood collection that ideally should be carried out anywhere and anytime [5, 6] so that no clinical visit and consultation of a health care provider is required. Blood sample collection needs to be easy to perform, painless, and provide samples of sufficient quality and quantity for reliable and rapid analysis. Successful implementation of such practice can potentially improve early detection and monitoring of disease [7], save labor and costs in clinical trials and practice [8–10], and enable flexible drug level monitoring [11]. Furthermore, self-laboratory results could be made available for clinical visits, thereby reducing potential delays in treatment decisions.

The most commonly used self-sampling method of the blood is the withdrawal of capillary blood from fingertips, which is perceived as painful and often provides insufficient sample quantities [12] for assessment of inflammation and autoantibodies. New blood sampling devices may have the potential to overcome these hurdles and optimize remote monitoring [12, 13]. We therefore tested the accuracy, feasibility, and acceptability of self-sampling of inflammation markers and autoantibodies in RA patients, comparing two forms of self-sampling of the capillary blood (upper arm and finger tip) with standard venous blood collection by health care professionals.

## Methods

### Study design and randomization

This study was a prospective, single-center, cross-sectional, parallel, two-group, non-blinded, randomized controlled trial (WHO International Clinical Trials Registry: DRKS00023526). The trial was approved by the local ethics authorities (Reg no. 320_20B) and designed in cooperation with three official patient partners (GB, MK, CE; Deutsche Rheuma-Liga Bundesverband e.V). Participants were consecutively recruited at the outpatient clinic of the Department of Internal Medicine 3 (FAU Erlangen-Nurnberg) between November 2020 and February 2021. To be included, patients had to fulfill the EULAR/ACR classification criteria for RA [14] and had to have antibodies against cyclic citrullinated peptides (anti-CCP) or rheumatoid factor (RF IgM).

**Fig. 1:**
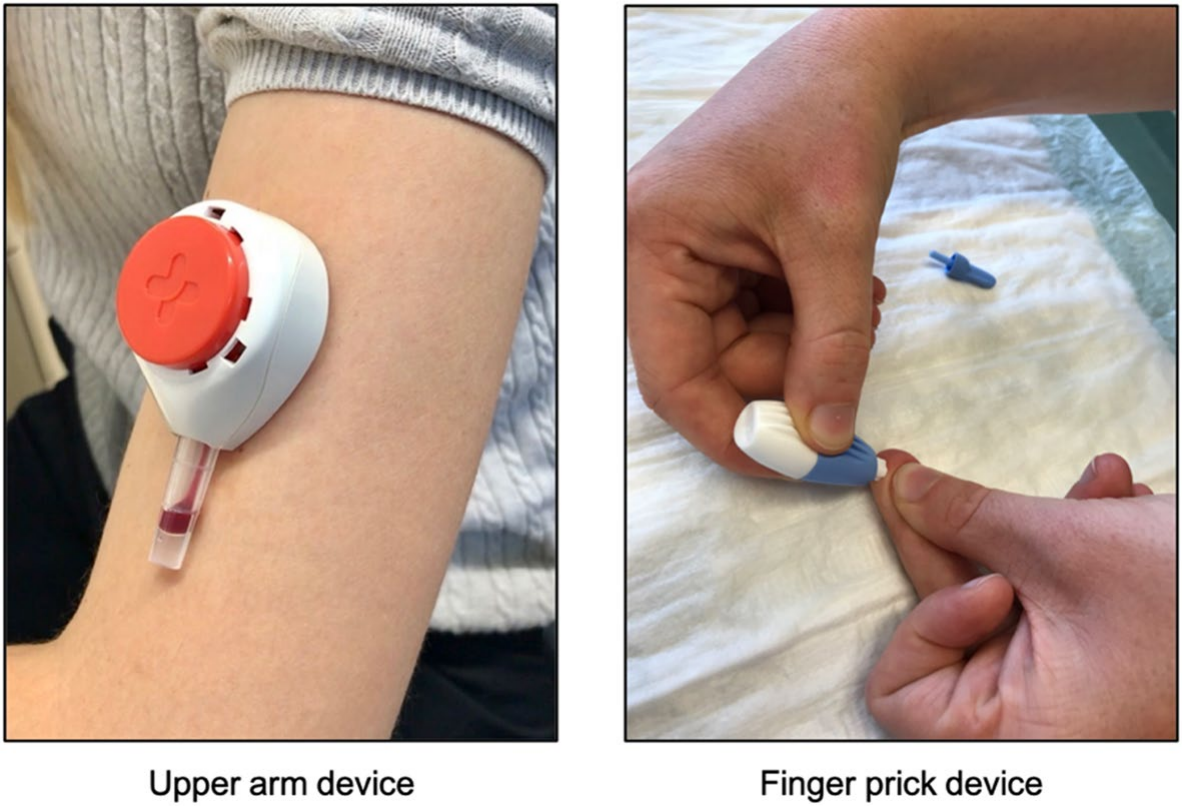
Capillary blood self-sampling devices. Left: Tasso SST^TM^ device used for upper-arm (UA) capillary blood sampling; right: BD Microtainer^TM^ finger prick device used in the fingertip sampling group (FP)

Participants were then randomly allocated in a 1:1 ratio to self-collection of the capillary blood via finger prick (BD Microtainer^TM^) (FP) or by a device designed for capillary blood collection from the upper-arm (via Tasso-SST^TM^ device) (UA). We used simple randomization, akin to random draws without replacement from a closed urn containing the planned total number of assignments. The randomization list was concealed by a member of the study team, who only made treatment allocations as per sequence when requested and was not otherwise involved in the trial. Patients in the FP group used traditional blue BD Microtainer^TM^ lancets (Becton, Dickinson and Company, blade diameters 1.5 mm (W) × 2.0 mm (D), Catalog No 366594, Franklin Lakes, NJ, USA) and BD SST^TM^ Microtainer blood collection tubes (Becton, Dickinson and Company, Catalog No 365967, Franklin Lakes, NJ, USA), allowing collection of up to approximately 1800 μl. After twisting off the safety cap, the lancet is activated by pressing against the finger in the FP group. Subsequently, the user needs to massage the finger and collect the capillary blood into a separate collection tube. Patients in the UA group used a Tasso-SST^TM^ device (Tasso Inc., Seattle, WA, USA), which is a self-adhesive lancet-based device to be used on the upper arm instead of the fingertip (Fig. 1). The device is attached to the upper arm by an adhesive, and the lancet is activated by pressing a button. Upon skin puncture, the device applies a vacuum to increase capillary blood flow. The blood is automatically collected into the attached tube with a maximum capacity of approximately 500 μl. The researchers who recruited and screened participants for eligibility were kept blinded to the randomization status. All patients provided written informed consent to participate in the study.

### Sample collection

Patients were instructed on how to use the self-sampling devices by local health care professionals and asked as to whether they had previously used self-sampling devices (for example for diabetes). In the UA group, written instructions were provided and a publicly available instruction video was presented by muting the original audio track in English, while the instructions displayed in the video were simultaneously explained in German. In the FP group, only oral instructions were given in German. Instructions included the sampling procedure, common pitfalls and how to avoid them. The primary instruction was to fill up the blood sampling containers within a maximum of 5 min. Patients were free to collect the samples from the dominant or nondominant arm. Assistance was available upon request, and study personnel recorded reasons for assistance. The time elapsed from skin puncture to retraction of blood collection device was recorded. Matched venous blood samples were obtained by trained phlebotomists from all participants within 1 h of capillary blood collection. The blood samples were kept at room temperature for at least 30 min, centrifuged for 15 min at 3200 RPM, and stored at 4 °C for 7–14 days. Samples were shipped to Thermo Fisher Scientific laboratory in Freiburg, Germany. Samples were inspected independently by two experienced lab technicians for quality. Upon arrival in the laboratory, the serum was transferred into Sarstedt™ 2 mL Polypropylene Micro Tubes (Sarstedt AG & Co., Nümbrecht, Germany) and stored at −20 °C until analysis. RF-IgM (EliA™ RF IgM, Thermo Fisher Scientific, Germany) and CCP-IgG antibodies (EliA™ CCP, Thermo Fisher Scientific, Freiburg, Germany) were analyzed on the Phadia^TM^ 250 instrument, and CRP was analyzed using Thermo Scientific™ Indiko™ Plus Clinical Chemistry Analyzer, Dreieich, Germany, once all samples were collected. While RF-IgM and CCP-IgG antibodies required only 5 μl of serum because the lab prepared manual dilutions, CRP measurement additionally required at least 100 μl of serum, so that CRP values were only available for samples with at least 105 μl of serum volume.

### Outcome measures

The primary outcome was the agreement of RF-IgM, CCP-IgG antibody, and CRP levels between matched capillary and venous samples. Secondary outcomes included pain perception, usability, general acceptance, and number of attempts for sampling. Volume and duration of the sample collection were also assessed for UA, FP, and venous blood sampling (VBS). Pain perception of capillary blood sampling and venipuncture was measured using a numeric pain rating-scale (NRS; 0 no pain at all, 10 worst imaginable pain) [15] directly after blood collection. Capillary sample volume and sample acquisition time were recorded as the time between perforation of the skin and closing of collection tube (maximum of 5 min). The usability was assessed with the validated System Usability Scale (SUS) [16].

Based on the adjective SUS rating scale as described by Bangor et al. [17], these values were translated to categories such as “excellent.” Acceptability was assessed using the Net Promoter Score (NPS) [18]. The NPS [18] asks patients how likely they are to recommend something to a friend or patient. Participants answer using a 11-point numeric rating scale (0-not at all likely to 10-extremely likely). Answers between 0 and 6 are summarized as detractors, 7–8 as passives, and 9–10 as promoters. The NPS is calculated by subtracting the percentage of detractors from the percentage of promoters. Finally, we assessed the proportion of patients willing to apply self-sampling at home and stating that they clearly understood when the self-sampling process was finished using the respective device.

### Statistical analysis

The sample size for this pilot study was based on convenience, and no formal sample-size calculation was undertaken. We summarized study group characteristics using appropriate summary statistics. We estimated intraclass correlation coefficients with 95% confidence intervals to quantify the agreement of analysis results obtained by venous and capillary sampling both overall and stratified by study groups. We also estimated and plotted Bland-Altman limits of agreement. Since the distributions of CCP, RF-IgM, and CRP tend to be lognormal, in addition to the conventional limits of agreement, we also estimated multiplicative limits of agreement using log differences that correspond to the ratio of values obtained from the venous sample and the capillary sample. Using the standard deviation of these log differences, limits of agreement were calculated separately for each mean value of capillary and venous sample results, based on the method described by Euser et. al. [19]. Mann-Whitney’s *U* test was used to compare continuous variables and scores for acceptability and pain in the two study arms and Wilcoxon’s signed-rank test for comparing pain scores between capillary and venous blood collection. Categorical variables were compared using the Fisher’s exact test. The level of significance was set as two-tailed *p* < 0.05 for all statistical tests. Analyses were carried out using the R software environment (version 4.0.1; R Foundation for Statistical Computing, Vienna, Austria).

## Results

### Participants

**Fig. 2:**
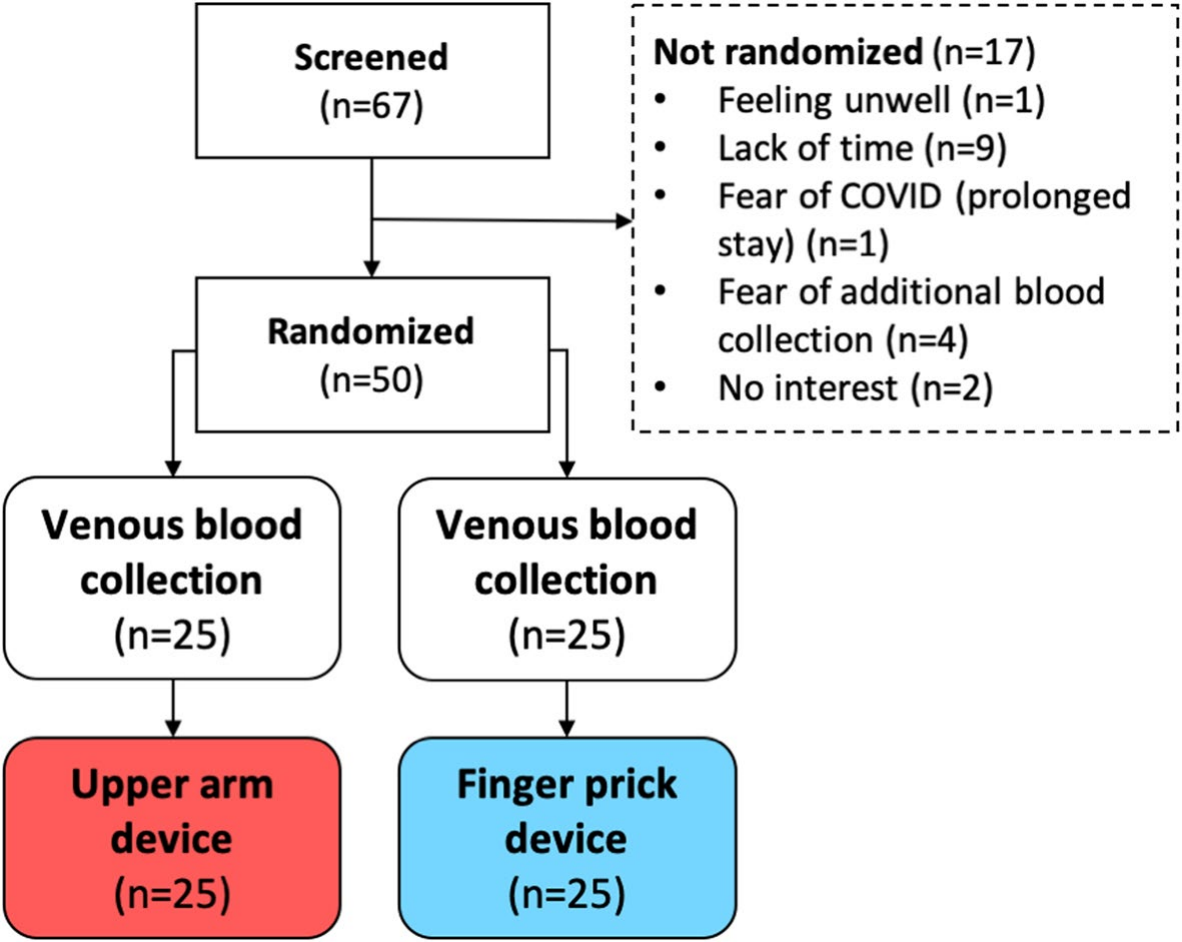
Patient flow diagram. Depiction of the number of screened rheumatoid arthritis patients, the number and reasons for screening failure, and the number of patients randomized to the two groups

A total of 67 seropositive RA patients were screened for eligibility, and 50 patients were randomized (Fig. 2). Demographic and disease characteristics are summarized in Table 1.

**Table 1:** Patient demographics and disease characteristics

Parameter	Upper arm (*n* = 25)	Finger prick (*n* = 25)	Total (*n* = 50)
Age, years, mean ± SD	56.7 ± 10.3	59.4 ± 13.9	58.0 ± 12.2
Age, years, median (range)	60.0 (36-72)	64.0 (31-80)	60.5 (31-80)
Female, *n* (%)	22 (88.0)	20 (80.0)	42 (84.0)
BMI, kg/m^2^, mean ± SD	26.7 ± 5.7	26.9 ± 5.5	26.8 ± 5.5
RF (positive), *n* (%)	21 (84.0)	20 (80.0)	41 (82.0)
ACPA (positive), *n* (%)	20 (80.0)	21 (84.0)	41 (82.0)
CRP, mg/l, mean ± SD	4.9 ± 13.0	6.8 ± 19.9	5.8 ± 16.7
Previous use of self-sampling device, *n* (%)	8 (32.0)	4 (16.0)	12 (48.0)
Swollen joint count, mean ± SD	1.1 ± 1.6	1.1 ± 2.1	1.1 ± 1.8
Tender joint count, mean ± SD	2.2 ± 2.8	1.2 ± 2.2	1.7 ± 2.5
PGA 0-10, mean ± SD	2.6 ± 2.6	1.8 ± 2.6	2.2 ± 1.3
DAS28-CRP, mean ± SD	2.4 ± 1.2	2.0 ± 1.4	2.2 ± 2.6

*ACPA* anti-citrullinated protein antibodies, *BMI* body mass index, *CRP* C-reactive protein, *DAS28* disease activity score, *PGA* patient global disease activity assessment, *RF* rheumatoid factor

### Capillary blood sample collection quality and procedure

In total, 49 matched capillary and venous samples were obtained, providing enough volume for the analysis of CCP and RF in 49 participants and additionally CRP in 22 participants (Figure S1). In the UA group, one patient was not able to collect blood despite attempting with a second device. In both groups, 4/25 (16%) of the patients were able to collect samples only in a second attempt (using a new self-sampling device) due to initial failure. In UA and FP groups, 8/25 (32%) and 7/25 (28%), respectively, requested assistance from the study personnel to perform the blood collection. The mean blood volume collected for UA and FP was 106.2 ± 60.6 μl and 118.8 ± 74.7 μl, respectively. The mean blood collection time for UA and FP was 256.8 ± 79.1 s and 230.6 ± 78.3 s, respectively.

### Agreement of anti-CCP Ab, RF-IgM, and CRP results between capillary and venous blood samples

**Fig. 3:**
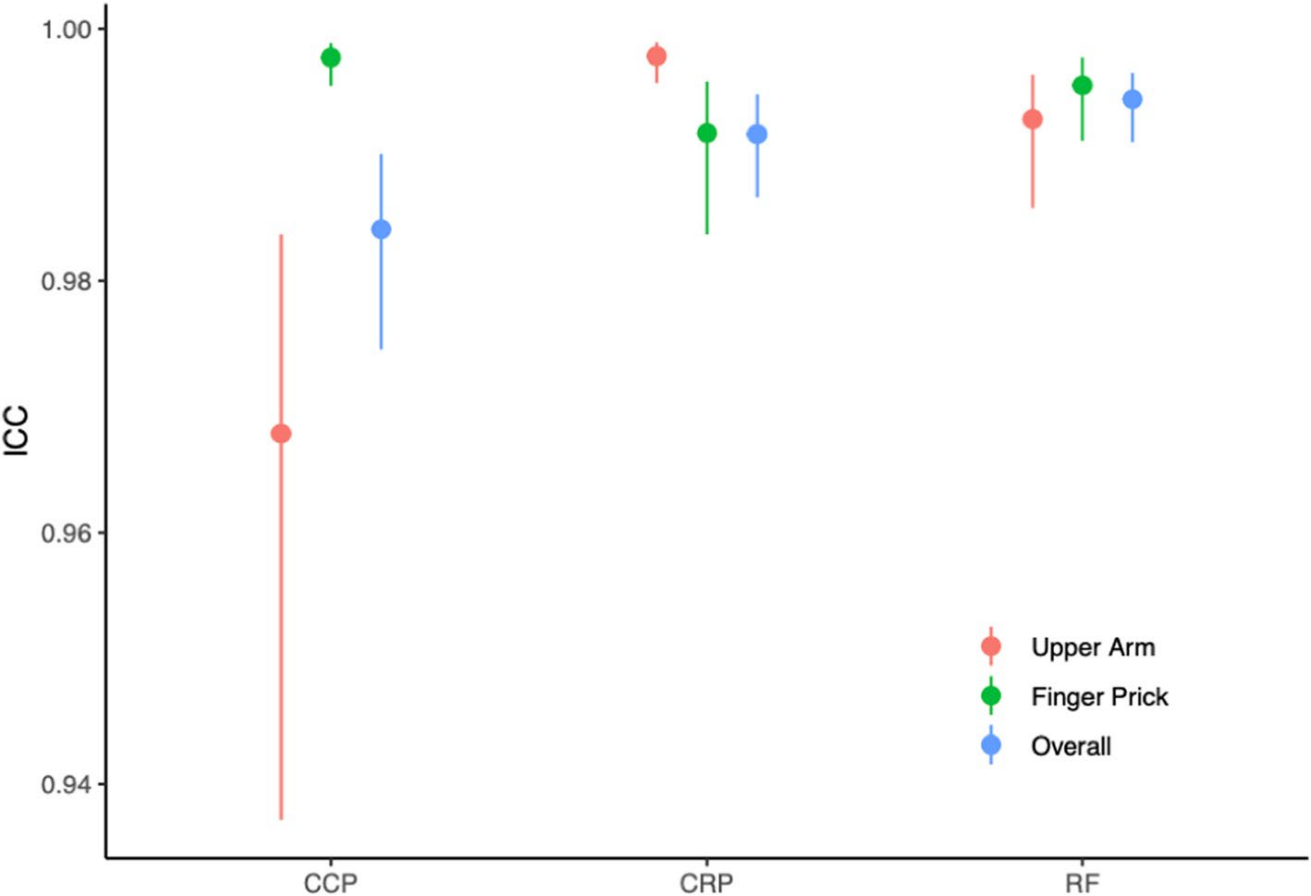
Agreement between capillary and venous blood sampling with respect to C-reactive protein and autoantibody results. Dots show the intraclass correlation coefficients, lines the 95% confidence intervals between self-sampling of the capillary blood from the upper arm (red) or via finger pricking (green) and venous blood sampling. Blue bars show the combined results of the upper arm and finger prick sampling. CCP, anti-cyclic citrullinated peptide antibodies; RF, rheumatoid factor immunoglobulin M; CRP, C-reactive protein

**Fig. 4:**
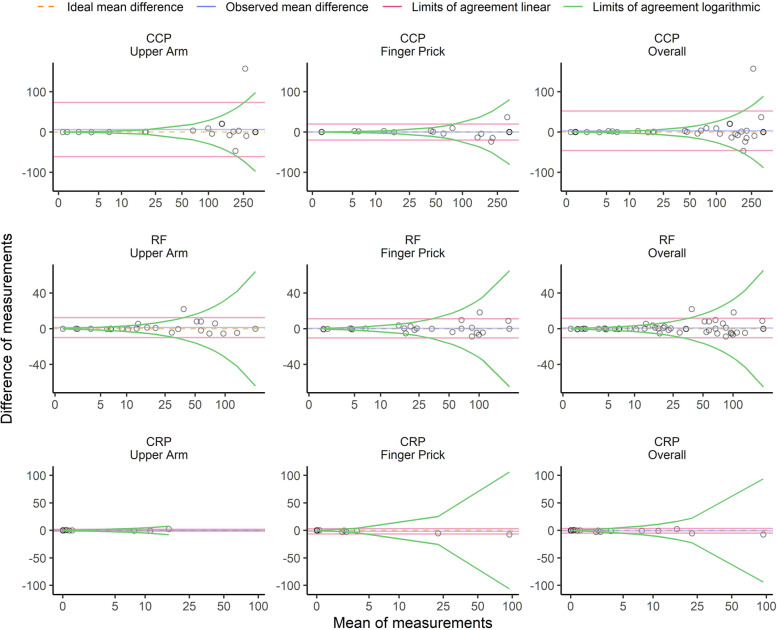
Bland-Altman comparison of the capillary blood and venous blood results with respect to C-reactive protein and autoantibody results. Bland-Altman diagrams showing differences in the measurements of anti-cyclic citrullinated peptide antibodies (CCP; U/ml), rheumatoid factor immunoglobulin M (RF; IU/ml)), and C-reactive protein (CRP; mg/l) between venous blood sampling and capillary blood sampling (upper arm, left; finger pricking, middle; combined groups, right). The dotted lines represent the ideal mean difference, the blue lines represent the observed mean difference, and the red and green lines indicate the limits of agreement on the additive scale and multiplicative scale, respectively

We observed excellent agreement between capillary and venous analyses. Intraclass correlation coefficients (95% CI) were high for anti-CCP (0.968; 0.937 to 0.984), RF (0.993; 0.986 to 0.996), and CRP (0.998; 0.996 to 0.999) in the UA group (Fig. 3 and Figure S2). Intraclass correlation coefficients (95% CI) were also high for anti-CCP (0.998; 0.995 to 0.999), RF (0.996; 0.991 to 0.998), and CRP (0.992; 0.984 to 0.996), in the FP group (Fig. 3 and Figure S2). The Bland-Altmann limits of agreement are shown in Fig. 4.

### Usability, pain, and acceptance of self-sampling

In the UA sampling group, 17/25 (68%) of the participants and in the FP sampling group 21/25 (84%) of the participants (*p*=0.32 by exact test) “agreed” or “completely agreed” that the respective device was easy to use. Mean SUS scores were 83.1 and 80.7 for UA and FP, respectively, translating to “excellent” usability using the method described by Bangor et al. [17] (Table 2). We did not observe substantial differences between the groups in individual item or total scores. Furthermore, 24/25 (96%) of patients in the UA group and 25/25 (100%) in the FP group stated that the instructions were clear. The end to blood collection was clearly discernible for 16/25 (64%) participants in the UA group and 19/25 (76%) in the FP group.

**Table 2:** Means and standard deviation scores for the System Usability Scale

Questions^**a**^	Upper arm (*n* = 25)	Finger prick (*n* = 25)	*P* value
I think I would like to use the system frequently, mean ± SD	4.0 ± 1.1	3.6 ± 1.5	0.30
I found the system to be unnecessarily complex	1.3 ± 0.6	1.9 ± 1.3	0.06
I thought the system was easy to use	4.0 ± 1.3	4.2 ± 1.2	0.46
I think that I would need support of a technical person to be able to use the system	1.6 ± 1.2	1.6 ± 1.2	0.61
I found the various functions in the system were well integrated	4.5 ± 1.0	4.0 ± 1.2	0.07
I thought there was too much inconsistency in the system	1.8 ± 1.4	1.7 ± 1.3	0.78
I would imagine that most people would learn to use the system very quickly	4.3 ± 1.0	4.3 ± 1.1	0.54
I found the system very cumbersome to use	1.7 ± 1.1	1.7 ± 1.3	0.87
I felt very confident using the system	4.3 ± 0.8	4.3 ± 1.2	0.35
I needed to learn a lot of things before I could get going with the system	1.4 ± 0.7	1.3 ± 0.7	0.16
System Usability Scale total score (out of 100)	83.1 ± 13.9	80.7 ± 20.5	0.98

^a^Responses were scored on a 5-point Likert scale: 1=strongly disagree, 5=strongly agree

**Fig. 5:**
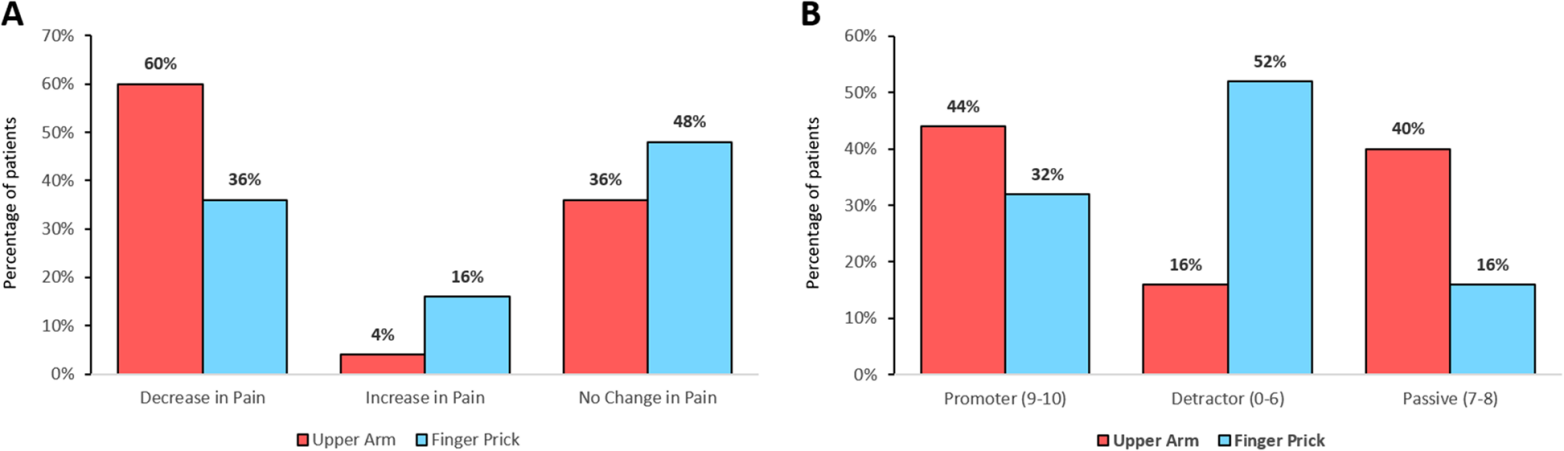
**A** Percentage of patients per group for respective change in pain and **B** percentage of patients per group for respective promoter score category

The mean pain NRS (range 0–10) scores for capillary self-sampling (UA: 1.7 ± 1.1; FP: 1.9 ± 1.9, *p*=0.93) were similar between the groups (*p*=0.93) and significantly lower than that in the standard venous blood collection (2.8 ± 1.7 in UA and 2.1 ± 2.0 in the FP group, *p*=0.003 overall). Numerically more patients in the UA group compared to the FP group experienced self-sampling as less painful compared to standard venous blood collection, 15/25 (60%) vs 9/25 (36%), see Fig. 5. The proportion of promoters was 11/25 (44%) in the UA group vs 8/25 (32%) in the FP group while 4/25 (16%) vs 13/25 (52%) were detractors, respectively (exact *p*=0.016 for all categories), resulting in a positive NPS for the UA group of +28% vs a negative NPS of −20% for the FP group, see Fig. 5. 15/25 (60%) and 8/25 (32%) stated that they would like to independently collect the capillary blood at home instead of seeing a professional for a venous blood collection in the UA and FP group, respectively.

## Discussion

This study showed excellent agreement of RF IgM, anti-CCP IgG, and CRP measurements between the capillary blood obtained from self-sampling and venous blood obtained by health care professionals in RA patients. The usability of both self-sampling devices was rated excellent by the participants. Most importantly, patients experienced less pain compared to traditional venous blood collection and upper-arm based self-sampling appears to be preferred over finger pricking by RA patients.

Laboratory results are essential to monitor disease activity of RA and to allow early detection of the disease [20]. Adding patient-centered self-sampling of the blood to already existing digital symptom self-assessment devices could therefore significantly increase the potential to monitor RA patients in a so far unprecedented way [7, 21, 22]. Furthermore, it can be expected that self-sampling could empower patients allowing them to time-independently obtain laboratory results that in case of CRP levels allow to objectify disease activity and potentially also to predict flares [23, 24]. While this approach is new to rheumatology, Pedersen and colleagues reported that self-monitoring (weekly questionnaire completion + fecal calprotectin test) using a web application helped to personalize treatment for patients suffering from ulcerative colitis [25] and Crohn’s disease [26]. In a recent feasibility trial patient self-sampling helped to substantially improve urate levels in patients with gout [27].

Our results are supported by a recent study that evaluated the perception, painfulness, and usability of the fingertip versus upper arm capillary blood collection among national athletes from Denmark [12]. Although another Tasso upper-arm device was used in the study, the self-sampling procedure and withdrawal mechanism are highly comparable. The associated pain using the upper arm device was rated lower compared to finger pricking (−0.4 ± 1.6, *p* < 0.05). Interestingly, the large majority (96%) of the athletes preferred capillary-based blood collection over venous blood collections and the majority (78%) preferred collection from the upper arm over the fingertip. In addition, also Blicharz and colleagues reported a significantly lower pain score associated with a similar upper arm device (TAP; Seventh Sense Biosystems, Medford, USA) compared to venous blood collection (0.4 vs 1.5 on pain scale from 0 to 10) investigating 143 participants [13]. A recent study evaluating the same upper-arm device also showed high correlation between venous and capillary blood when testing for anti-SARS-CoV-2 antibodies [28].

The upper-arm device failure rate was higher in our study compared to previous studies performed with the same device (20% (6/30) vs 2% (2/108) [12] and 4% (10/240) [29]). A main reason for this difference could be the fact that capillary blood collection in our study was carried out by patients themselves and not by healthy individuals or health care professionals. Our study collective had RA and therefore was to some extent handicapped by the disease. Indeed, self-collection of the blood in the UA group was impaired as patients were requesting help to press the button (5/25; 20%) and to remove the blood collection container (3/25; 12%). Using the same upper-arm device, healthy participants were able to successfully draw blood more often during the first draw and without assistance in a previous study [28] (first draw: 93 vs 80%; second draw 7 vs 16%). Future studies should analyze the failure rate longitudinally, as experienced users might use these devices more successfully. Additional to this aspect, we are currently prospectively investigating self-sampling feasibility in different rheumatic patient populations, at home scenarios and healthy individuals, also including qualitative patient feedback.

Similar to the aforementioned athlete study, RA patients did attest UA sampling a higher usability (SUS) than FP sampling. However, when plotting our SUS results on an adjective rating scale as described by Bangor et al. [17], SUS scores for both devices were rated “excellent”. Also, acceptance was higher for the UA than for FP sampling (NPS +28% vs. −20). Furthermore, the majority of UA patients (60% compared to 32% in FP group) stated that they would like to independently collect capillary blood at home instead of seeing a healthy professional for a venous blood collection. Offering patients a variety of blood collection sites is likely to decrease pain and discomfort. Regarding patients with veins difficult to detect even for health care professionals, patients with fear of needles, bruising, and general anxiety, capillary-based self-sampling of the blood may be a good alternative.

The parallel group design is a limitation of this study. Patients could have used both the UA and the FP device sequentially allowing a direct comparison; however, the sequence of device use may have also caused priming and bias in patient-reported outcomes. We have therefore deliberately chosen a parallel group design, which has also allowed us to reduce the number of blood collections. Furthermore, the limited volume of the blood obtained by the capillary collection devices impeded the analysis of CRP in a number of patients. This is a limitation of capillary blood sampling requiring further development in device design. Simulation of a an “at-home” situation and manual work needed to analyze probes are further limitations of this study. On the other hand, patient involvement in the study design and in conducting the study represents strengths of this study. In addition, the capillary self-sampling devices were used by patients themselves and not by experienced health care professionals, as in previous studies [12, 13, 29].

To our knowledge, this is the first rheumatology study that has performed a structured analysis on self-sampling of the capillary blood for the measurement of inflammation markers and autoantibodies. Accuracy, feasibility, and acceptance of the two self-sampling devices were high providing the possibility for remote analysis. While more studies are needed to effectively implement this novel technology in disease monitoring, our data provide evidence for the principal feasibility of such an approach. Considering the higher acceptance of UA sampling, future research should focus on analyzing such devices, i.e., the standardization of self-sampling procedures such as application of chemical heat pads [29] to improve blood circulation in the skin and blood volume output. Furthermore, online counseling [30, 31] could guarantee the assistance that was necessary in some of the patients.

## Conclusion

The excellent usability and high concordance with the results from venous blood analysis illustrate the potential of capillary blood self-sampling. This approach does not only provide more comfort, higher flexibility, and less pain but also supports tight disease monitoring and potentially also improves the early recognition of RA.

## Supplementary Information

**Additional file 1: Fig. S1.** Self-sampling feasibility results according to randomization arm

**Additional file 2: Fig. S2.** Intraclass correlation coefficients and 95% confidence interval by group and analyte

## Data Availability

Data analyzed during the current study are available from the corresponding author on reasonable request.

## Abbreviations

ACR: American College of Rheumatology
CCP: Cyclic citrullinated peptides
CRP: C-reactive Protein
EULAR: European alliance of associations for rheumatology
FP: Finger prick
NPS: Net Promoter Score
NRS: Numeric rating scale
RA: Rheumatoid arthritis
RF: Rheumatoid factor
SUS: System Usability Scale
UA: Upper-arm
VBS: Venous blood sampling
