# Supplementary material for: Accuracy and tolerability of self-sampling of capillary blood for analysis of inflammation and autoantibodies in rheumatoid arthritis patients—results from a randomized controlled trial
Source: Arthritis Res Ther. 2022 May 25;24:125. doi: 10.1186/s13075-022-02809-7 (PMC9130452; doi:10.1186/s13075-022-02809-7)
Supplement: Supplementary file 1 — Additional file 1: Fig. S1. Self-sampling feasibility results according to randomization arm [file 13075_2022_2809_MOESM1_ESM.pdf]

Self-sampling feasibility results according to randomization arm

**Variable**	**Upper Arm** (n = 25)	**Finger Prick** (n = 25)
Patients succeeding at first attempt, n (%)	20 (80.0)	21(84.0)
Patients succeeding at second attempt, n (%)	4 (16.0)	4 (16.0)
Patients not succeeding at all, n (%)	1 (4.0)	0 (0.0)
Patients requiring assistance, n (%)	8 (32.0)	7 (28.0)
Reasons for assistance[a] , n		
Opening kit	3	x
Pressing button	5	x
Removing button cap (UA) / twisting off the tab (FP)	1	6
Removing collection tube	3	x
Collecting blood	x	3
Volume of blood collected (µl), mean ± SD	106.2 ± 60.6	118.8 ± 74.7
Patients with sufficient Blood for analysis of all three biomarkers, n (%)	10 (40.0)	12 (48.0)

[a]Multiple reasons could be recorded per patient
