# Supplementary material for: Accuracy and tolerability of self-sampling of capillary blood for analysis of inflammation and autoantibodies in rheumatoid arthritis patients—results from a randomized controlled trial
Source: Arthritis Res Ther. 2022 May 25;24:125. doi: 10.1186/s13075-022-02809-7 (PMC9130452; doi:10.1186/s13075-022-02809-7)
Supplement: Supplementary file 2 — Additional file 2: Fig. S2. Intraclass correlation coefficients and 95% confidence interval by group and analyte [file 13075_2022_2809_MOESM2_ESM.pdf]

Intraclass correlation coefficients and 95% confidence interval by group and analyte

| Group | CCP | CRP | RF |
|---|---|---|---|
| Upper Arm | 0.968 (0.937 - 0.984) | 0.998 (0.996 - 0.999) | 0.993 (0.986 - 0.996) |
| Finger Prick | 0.998 (0.995 - 0.999) | 0.992 (0.984 - 0.996) | 0.996 (0.991 - 0.998) |
| Overall | 0.984 (0.975 - 0.990) | 0.992 (0.987 - 0.995) | 0.994 (0.991 - 0.997) |
